# Chitin perception in plasmodesmata characterizes submembrane immune-signaling specificity in plants

**DOI:** 10.1073/pnas.1907799117

**Published:** 2020-04-13

**Authors:** Cécilia Cheval, Sebastian Samwald, Matthew G. Johnston, Jeroen de Keijzer, Andrew Breakspear, Xiaokun Liu, Annalisa Bellandi, Yasuhiro Kadota, Cyril Zipfel, Christine Faulkner

**Affiliations:** ^a^Crop Genetics, John Innes Centre, NR4 7UH Norwich, United Kingdom;; ^b^The Sainsbury Laboratory, University of East Anglia, NR4 7UH Norwich, United Kingdom;; ^c^Institute of Plant and Microbial Biology, University of Zürich, CH-8008 Zürich, Switzerland;; ^d^Zürich-Basel Plant Science Centre, University of Zürich, CH-8008 Zürich, Switzerland

**Keywords:** membrane domain, receptor signaling, plasmodesmata, immunity

## Abstract

The plasma membrane (PM) is composed of heterogeneous subdomains, characterized by differences in protein and lipid composition. PM receptors can be dynamically sorted into membrane domains to underpin signaling in response to extracellular stimuli. In plants, the plasmodesmal PM is a discrete microdomain that hosts specific receptors and responses. We exploited the independence of this PM domain to investigate how membrane domains can independently integrate a signal that triggers responses across the cell. Focusing on chitin signaling, we found that responses in the plasmodesmal PM require the LysM receptor kinases LYK4 and LYK5 in addition to LYM2. Chitin induces dynamic changes in the localization, association, or mobility of these receptors, but only LYM2 and LYK4 are detected in the plasmodesmal PM. We further uncovered that chitin-induced production of reactive oxygen species and callose depends on specific signaling events that lead to plasmodesmata closure. Our results demonstrate that distinct membrane domains can integrate a common signal with specific machinery that initiates discrete signaling cascades to produce a localized response.

## Significance

Plasmodesmata connect the cytoplasm of neighboring plant cells across cell walls. In response to various signals, they open and close to connect and isolate cells. We have found that the plasmodesmal plasma membrane hosts a unique immune-signaling cascade—different from that in the surrounding plasma membrane—which leads to plasmodesmata closure and cell isolation upon chitin perception. This response is mediated by a specific receptor complex, which in turn activates an NADPH oxidase via a specific regulatory module. This work characterizes how a cell can produce a localized and specific response in a discrete membrane domain, identifying that there is microdomain specificity in immune signaling to a single elicitor and that cell-to-cell connections are independently controlled.

An array of plasma membrane (PM)-localized receptors projects into the extracellular environment to perceive a multitude of molecular signals. Receptor proteins (RPs) and receptor kinases (RKs) commonly act in complexes to activate signaling cascades upon binding their cognate ligands, mediating responses to a range of developmental and environmental signals. Plant cells exploit these receptors to perceive and signal in response to the presence of microbes, making these receptors critical components of the innate immune system. Thus, the mechanisms of receptor activation and signaling are central to plant cellular responses.

Across kingdoms, the PM is compartmentalized into subdomains diverse in composition, function, and dynamics. These subdomains regulate the organization and activation of a variety of proteins resident within them and thus define specific cellular responses. Some RKs and RPs dynamically form modular receptor complexes in PM domains (micro- or nanodomains) to establish signaling hubs that execute response outputs (1–3). This is observed in animal immune signaling: receptor activation is conferred by membrane domain association in examples such as lipopolysaccharide (LPS) signaling and B cell activation (4–7). Similarly, in plants, receptor association with membrane domains can define signaling. For example, the *Medicago* LysM-CONTAINING RECEPTOR-LIKE KINASE 3 (LYK3) is recruited to a PM microdomain during rhizobia infection (8), and the flg22 receptor FLAGELLIN SENSING 2 (FLS2) is stabilized in nanodomains during signaling (9).

Membrane microdomains can be associated with specific subcellular structures. For example, in plants, the plasmodesmal PM lines plasmodesmata (singular plasmodesma), the tubes that bridge neighboring cells to establish the interconnected cytoplasm. While electron micrographs identify that the plasmodesmal PM is continuous with the PM, the protein and lipid composition of the plasmodesmal PM differs from the rest of the PM (10–12), identifying it as a discrete PM microdomain. Plasmodesmal aperture is regulated by the synthesis and hydrolysis of the β-1,3-glucan callose (13–15), and the enzymes and regulators that control this are anchored in the plasmodesmal PM (15–18), suggesting plasmodesmal function is underpinned by specificity of the plasmodesmal PM microdomain. This is further supported by increasing examples of receptors that are specifically located at, or active in, the plasmodesmal PM. For example, the *Arabidopsis* CRINKLY4 (ACR4) and CLAVATA1 (CLV1) RKs form plasmodesmata-specific complexes (19), and, recently, several RKs have been shown to be dynamically recruited to the plasmodesmal PM in response to stress (20, 21). While the significance of this is not yet known, it implies specific machinery can be recruited to the plasmodesmal PM to execute signaling.

In the context of microbial perception and immune signaling, we have previously identified specific machinery required for plasmodesmal responses: the LYSM-CONTAINING GPI-ANCHORED PROTEIN 2 (LYM2) is located in the plasmodesmal PM and mediates chitin-triggered plasmodesmal closure (22), and the CALMODULIN-LIKE 41 protein mediates flg22-triggered plasmodesmal closure (16). Significantly, LYM2 functions independently of the CHITIN ELICITOR RECEPTOR KINASE (CERK1) chitin receptor that mediates other chitin-induced signals in the PM (22).

It is not clear how an independent signaling cascade is executed in the plasmodesmal PM distinct from simultaneous PM signaling. Here, we exploited the specific involvement of LYM2 in chitin-triggered plasmodesmal responses to investigate the mechanisms by which neighboring membrane domains can signal independently. We found that in addition to LYM2, chitin responses in the plasmodesmal PM genetically require two additional LysM-RKs, LYK4 and LYK5. LYM2 can associate with both LYK4 and LYK5, but we detected only LYK4 in plasmodesmata, suggesting that chitin-triggered plasmodesmal signaling is mediated directly by a LYM2-LYK4 complex. The dependence of plasmodesmal responses on LYK5 appears to rest on its association with LYK4 in the PM prior to chitin perception. Chitin perception by LYM2 triggers activation of the NADPH oxidase RESPIRATORY BURST OXIDASE HOMOLOG PROTEIN D (RBOHD) via a specific phosphorylation signature linked to calcium-dependent protein kinases (CPKs). Supporting this, CPK6 and CPK11 are required to mediate chitin-triggered plasmodesmal responses. Ultimately, this signaling cascade induces callose deposition and plasmodesmata closure. Our findings characterize how the plasmodesmal PM specifically executes an immune-signaling cascade, illustrating how a single ligand can trigger independent responses in different membrane microdomains and demonstrating that plant cells can compartmentalize different outputs within the PM.

## Results

### Chitin-Triggered Plasmodesmata Closure Is Dependent on LYK4 and LYK5.

**Fig. 1.**
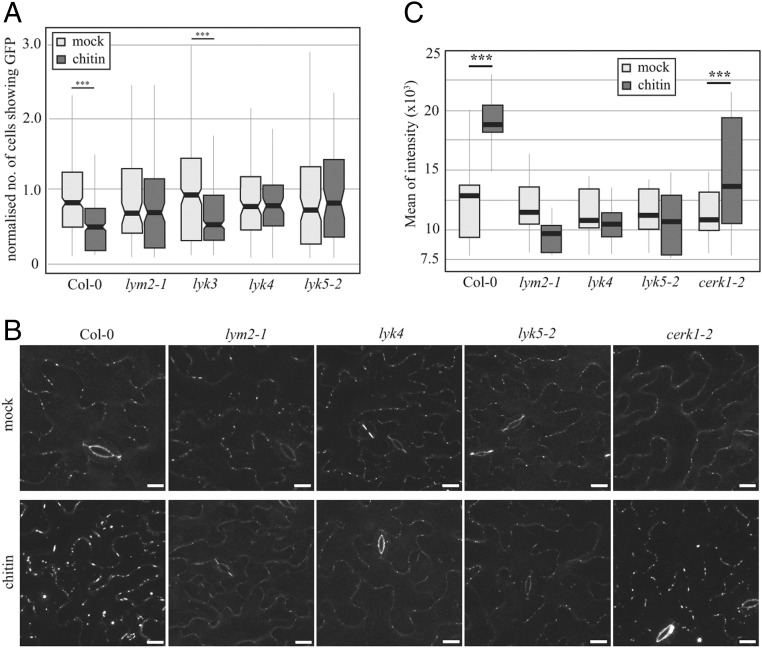
LYK4 and LYK5 regulate plasmodesmal permeability in response to chitin. (*A*) Microprojectile bombardment into leaf tissue of 5- to 6‐wk‐old *Arabidopsis* shows that Col-0 and *lyk3* but not *lym2-1*, *lyk4*, and *lyk5-2* exhibit reduced movement of GFP to neighboring cells in response to chitin. Data were collected from six biological replicates, and the number of cells showing GFP has been normalized to the mean of the mock-treated data within genotypes. These data are summarized in box plots in which the line within the box marks the median, the box signifies the upper and lower quartiles, and the whiskers represent the minimum and maximum within 1.5 × interquartile range. Notches represent approximate 95% confidence intervals. The number of bombardment sites (*n*) counted is ≥84. Asterisks indicate statistical significance compared with control conditions. ***P < 0.001. (*B*) Confocal images of aniline blue-stained plasmodesmal callose in leaves of 5- to 6‐week‐old Col-0 plants, as well as *lym2-1*, *lyk4*, *lyk5-2*, *and cerk1-2 mutants*. Images were acquired 30 min postinfiltration with water or chitin. (Scale bars, 15 µm.) (*C*) Quantification of plasmodesmata-associated fluorescence of aniline blue stained callose using automated image analysis. Col-0 and the *cerk1-2* mutant show an increase in aniline blue stained plasmodesmal callose 30 min post chitin treatment. In *lym2-1*, *lyk4*, and *lyk5-2*, this response is not detected. This correlates with the movement phenotype and identifies that chitin-triggered plasmdesmata closure is caused by callose deposition at plasmodesmata. The fluorescence intensity is summarized in box plots in which the line within the box marks the median, the box signifies the upper and lower quartiles, the minimum and maximum within 1.5 × interquartile range. Number of images (*n*) is ≥31, and *** indicates *P* < 0.001 when chitin-treated samples were compared with mock treatments within genotypes.

We previously identified LYM2 as a glycophosphatidylinositol (GPI)-anchored LysM-RP that resides in the plasmodesmal PM (22). Ligand perception by LysM-RKs and -RPs often involves multiple members of the LysM protein family (23–26), and LYM2 has no intracellular domains for signaling. Thus, we hypothesized that LYM2 might partner with a LysM-RK for signaling. The *Arabidopsis* LysM-RK family consists of five members: CERK1/LYK1, LYK2, LYK3, LYK4, and LYK5. To narrow down plasmodesmata signaling candidates, we performed RT-PCR to identify members of the family expressed in mature *Arabidopsis* leaves where LYM2 functions. Only transcripts from *CERK1*, *LYK3*, *LYK4*, and *LYK5* were detected in mature leaves (*SI Appendix*, Fig. S1). As we previously showed that CERK1 is not required for chitin-triggered plasmodesmata closure (22), we assayed for chitin-triggered plasmodesmal responses in *lyk3*, *lyk4* (27), and *lyk5-2* (23) knockout mutants. Microprojectile-bombardment assays, in which movement of GFP from single transformed cells within the *Arabidopsis* leaf epidermis is measured (22), showed that *lyk4* and *lyk5-2* mutants do not exhibit the chitin-triggered reduction in the cell-to-cell spread of GFP, indicative of plasmodesmata closure, observed in wild-type (Col-0) plants (Fig. 1 and *SI Appendix*, Fig. S2). Thus, LYK4 and LYK5 are required for chitin-triggered plasmodesmata closure and are candidate partners for LYM2-mediated signaling.

### LYM2-Dependent Chitin Signaling Induces Callose Deposition at Plasmodesmata.

Callose is a β-1,3-glucan polymer deposited at plasmodesmata during stages of development and in response to a range of stresses to induce plasmodesmal closure (15, 16, 18). Recently, we showed that callose is deposited at plasmodesmata in response to flg22 (16), and therefore we examined callose deposition at plasmodesmata in response to chitin to determine if this is common in different microbial signals. We quantified aniline blue-stained, plasmodesmata-associated callose deposits in chitin-treated and mock-treated leaf tissue of Col-0 plants, and, as for flg22 (16), plasmodesmata-located aniline blue fluorescence increased significantly within 1 h of chitin treatment (Fig. 1 *B* and *C*). Aniline blue staining of *cerk1-2* mutants also shows an increase in plasmodesmal callose deposition in response to chitin, consistent with the chitin-triggered reduction in GFP movement through plasmodesmata previously observed in this mutant (22). Staining of *lym2-1*, *lyk4*, and *lyk5-2* mutant leaves indicated that chitin does not trigger an increase in callose at plasmodesmata in these mutants. Thus, increased callose deposition negatively correlates with GFP spread, suggesting that callose deposition is the mechanism by which plasmodesmata close in response to chitin.

### LYK4, but Not LYK5, Is Present in Plasmodesmata.

Having identified that LYK4 and LYK5 are required for chitin-triggered plasmodesmata closure, we examined their subcellular localization to determine if they are present at plasmodesmata. We generated translational fusions of LYK4 and LYK5 to RFP and, as previously observed (27, 28), they localized to the PM in the absence and presence of chitin and showed no enrichment at plasmodesmata (*SI Appendix*, Fig. S3).

**Fig. 2.**
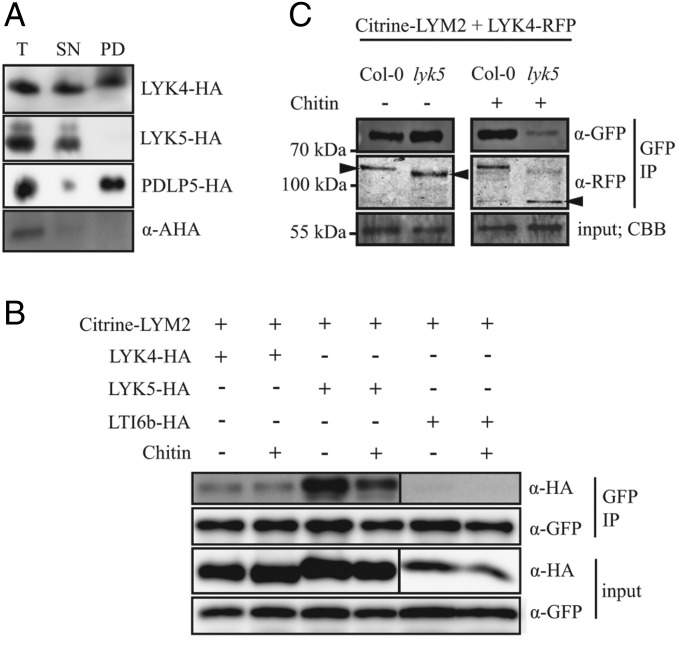
LYK4 and LYK5 can associate with LYM2 but only LYK4 is detected in plasmodesmata. (*A*) Western blot analysis of purified plasmodesmata fractions from *N. benthamiana* tissue expressing LYK4-HA, LYK5-HA, or PDLP5-HA. Total (T) (extracts from ground tissue), supernatant (SN) (all cellular material excluding cell walls), and plasmodesmatal (PD) (membranes released from purified cell walls) extracts were separated by SDS/PAGE and probed with anti-HA to determine the presence of LYK4-HA, LYK5-HA, and PDLP5-HA in each fraction or with anti–H^+^-ATPase (AHA) to detect PD-excluded H^+^-ATPases. (*B*) Western blot analysis of immunoprecipitated proteins from *N. benthamiana* tissue expressing Citrine-LYM2 and LYK4-HA, LYK5-HA, or LTI6b-HA. LYK4-HA and LYK5-HA are detected in detergent-extracted fractions by IP of Citrine-LYM2. Input and immunoprecipitated (IP) samples were probed α-GFP and α-HA antibodies as indicated. CBB, Coomassie brilliant blue. (*C*) Western blot analysis of immunoprecipitated protein extracts from *Arabidopsis* protoplasts expressing *Citrine-LYM2* and *LYK4-RFP*. LYK4-RFP is detected in samples from both Col-0 and *lyk5-2* protoplasts. Input and immunoprecipitated (IP) samples were probed with anti-GFP and anti-RFP antibodies as indicated. LYK4-RFP bands of different sizes are indicated by arrowheads; size markers are indicated to the left. (*A*–*C*) Experiments were repeated three times with similar results.

To further probe LYK4 and LYK5 association with plasmodesmata, we performed plasmodesmata purification and protein extraction (Fig. 2*A*). For this, we expressed LYK4-HA and LYK5-HA in *Nicotiana benthamiana* tissue and purified plasmodesmata. As a positive control, we used the plasmodesmal protein PDLP5-HA (29, 30), and as a negative control, we probed extracts with a polyclonal antibody that detects H^+^-ATPase transporters that are excluded from plasmodesmata (10, 31). We detected PDLP5-HA and LYK4-HA in purified plasmodesmata extracts, but not LYK5-HA or H^+^-ATPase (Fig. 2*A*), suggesting that only LYK4 is a plasmodesmal PM resident protein. Therefore, despite the genetic dependence of chitin-triggered plasmodesmata closure on *LYM2*, *LYK4*, and *LYK5*, these three receptors are not likely to act cooperatively within plasmodesmata.

### LYM2 Associates with LYK4 and LYK5 in the PM.

The dependence of chitin-triggered plasmodesmal closure on LYM2, LYK4, and LYK5, as well as the capacity for LysM receptors to interact with other family members, suggests that these receptors form complexes to mediate signaling. To determine if LYM2 can associate with LYK4 and LYK5 in the PM, we performed targeted coimmunoprecipitation (co-IP) assays. We coexpressed Citrine-LYM2 with LYK4-HA, LYK5-HA, or LTI6b-HA in *N. benthamiana* leaves and immunoprecipitated Citrine-LYM2 from detergent-extracted fractions (Fig. 2*B*). While LTI6b-HA did not coimmunoprecipitate with Citrine-LYM2, both LYK4-HA and LYK5-HA did from both water- and chitin-treated tissue. While this suggests that LYM2 can associate with LYK4 and LYK5 in a chitin-independent manner, we consistently observed that less LYK5-HA immunoprecipitated with LYM2 following chitin treatment. This raises the possibility that chitin perturbs the LYM2-LYK5 association. We also noted that less LYK4-HA immunoprecipitates with LYM2 than LYK5-HA, suggesting that LYM2-LYK4 association is less abundant in the PM.

Given the genetic dependence of plasmodesmal response on *LYK5*, but its absence from the plasmodesmata, we tested its role in the association between LYM2 and LYK4. For this, we transformed *Arabidopsis lyk5-2* protoplasts with Citrine-LYM2 and LYK4-RFP (Fig. 2*C*) and performed targeted co-IPs. We observed that LYK4-RFP immunoprecipitated with Citrine-LYM2 in a chitin-independent manner from both Col-0 and *lyk5-2* protoplasts, demonstrating that the association between LYM2 and LYK4 is LYK5-independent. However, we observed that LYK4-RFP migrates faster on sodium dodecyl sulfate/polyacrylamide gel electrophoresis (SDS/PAGE) gels when extracted from *lyk5-2* protoplasts and following chitin treatment the dominant LYK4-RFP band detected from *lyk5-2* protoplasts is ∼30 kDa smaller than the dominant band in the Col-0 protoplasts (Fig. 2*C*). This suggests that LYK4 is modified or stabilized in a LYK5-dependent manner, which might explain the critical role for LYK5 function in plasmodesmal responses.

### LYK4 and LYK5 Associate in the PM.

**Fig. 3.**
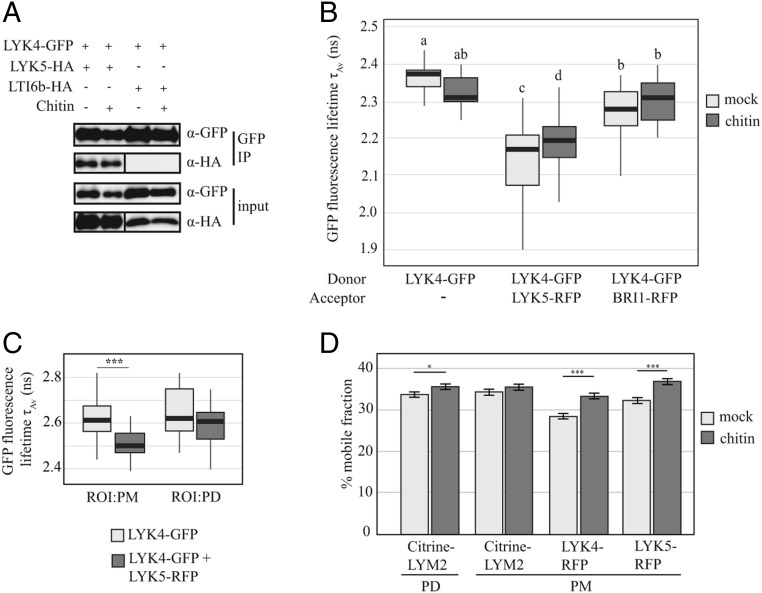
LYK5 dynamically associates with LYK4 in the PM. (*A*) Western blot analysis of immunoprecipitated (IP) protein extracts from *N. benthamiana* tissue expressing LYK4-GFP and LYK5-HA or LTI6b-HA. LYK4-GFP was immunoprecipitated from detergent-extracted fractions and probed with anti-GFP and anti-HA to detect LYK4-GFP and LYK5-HA or LTI6b-HA, respectively. Experiments were repeated three times with similar results. (*B*) FRET-FLIM analysis of LYK4-GFP in the presence of acceptors LYK5-RFP or BRI1-RFP and the presence and absence of chitin. Fluorescence lifetime was measured in *N. benthamiana* tissue transiently coexpressing the indicated constructs as donors or acceptors. Box plots represent GFP fluorescence-weighted average lifetime (τ_Av_, ns): the line within the box marks the median, the box signifies the upper and lower quartiles, the whiskers represent the minimum and maximum within 1.5 × interquartile range. Data were analyzed by ANOVA with a post hoc Tukey multiple comparison of means (*P* value < 0.01). Samples with the same letter code are not significantly different. Number of images (*n*) analyzed is ≥19. (*C*) FRET-FLIM analysis of LYK4-GFP at the plasmodesmal PM and the PM in the presence and absence of LYK5-RFP in *N. benthamiana* tissue. Plasmodesmata were marked by coexpression of Citrine-LYM2 and ROI were defined around plasmodesmata (PD) and in the PM for analysis. Box-plots represent GFP fluorescence-weighted average lifetime (τ_Av_, ns): the line within the box marks the median, the box signifies the upper and lower quartiles, and the whiskers represent the minimum and maximum within 1.5 × interquartile range. Asterisks indicate statistical significance compared with control conditions (***P < 0.001). Number of ROIs (*n*) analyzed is ≥27. (*D*) % mobile fraction of LYM2, LYK4 and LYK5 as measured by FRAP assays. For Citrine-LYM2 FRAP measurements were taken for the plasmodesmata-located (PD) and PM-located pools of receptor. For LYK4-RFP and LYK5-RFP, FRAP measurements were taken in the PM. Error bars are SE. *P < 0.05; **P < 0.001. Number of FRAP experiments analyzed is ≥43.

To further explore the role of LYK5 in plasmodesmal signaling and its association with LYK4, we explored the possibility that LYK5 directly associates with LYK4 in the PM. Immunoprecipitation (IP) of LYK4-GFP from membrane fractions of *N. benthamiana* tissue coexpressing LYK5-HA or LTI6b-HA identified that LYK5-HA associates with LYK4-GFP in the PM in a chitin-independent manner (Fig. 3*A*). LTI6b-HA did not associate with LYK4-GFP. We further investigated the dynamics and localization of this association with Förster resonance energy transfer–fluorescence lifetime imaging (FRET-FLIM) analysis (Fig. 3*B*). The fluorescence lifetime (average amplitude [τ_Av_]) of PM-localized LYK4-GFP was significantly reduced in the presence of LYK5-RFP, indicating an increase in FRET as expected for interacting proteins (*SI Appendix*, Table S1). The change in FRET efficiency induced by chitin is ∼3.4%, suggesting that in this overexpression system, the pool of RKs that dissociate is small (and why it is undetectable in the Western blot). Chitin treatment decreased the LYK5-RFP–induced reduction in fluorescence lifetime of LYK4-GFP, indicating reduced FRET between these two proteins. To localize the occurrence of FRET within the PM, we marked plasmodesmata by coexpression of LYK4-GFP and LYK5-RFP with Citrine-LYM2. When we compared τ_Av_ of LYK4-GFP in regions of interest (ROIs) within the PM and at plasmodesmata, we observed that τ_Av_ in the PM was significantly reduced by the presence of LYK5-RFP but that in plasmodesmata, it was not (Fig. 3*C* and *SI Appendix*, Table S1). These data support our finding that LYK5 is not present in the plasmodesmal PM and further suggest that chitin weakens the interaction between LYK4 and LYK5 in the PM, possibly by complex dissociation or a change in complex conformation.

Changes in protein association might result in a change in mobility within the PM. To test this, we made fluorescence recovery after photobleaching (FRAP) measurements to quantify the mobile fraction of the receptor population in the absence and presence of chitin. The mobile fraction of LYK4-RFP and LYK5-RFP both increased significantly 60 s postbleaching in the presence of chitin relative to mock conditions (Fig. 3*D*). By contrast, chitin treatment did not change the mobile fraction of Citrine-LYM2 in the PM but marginally changed it at plasmodesmata, indicating that there is a small increase in the pool of receptor moving into the plasmodesmal ROI. The changes in the mobile fractions of LYK4 and LYK5 further support a model in which a pool of LYK4 and LYK5 change their associations in response to chitin.

### LYM2 Accumulates at Plasmodesmata in Response to Chitin.

**Fig. 4.**
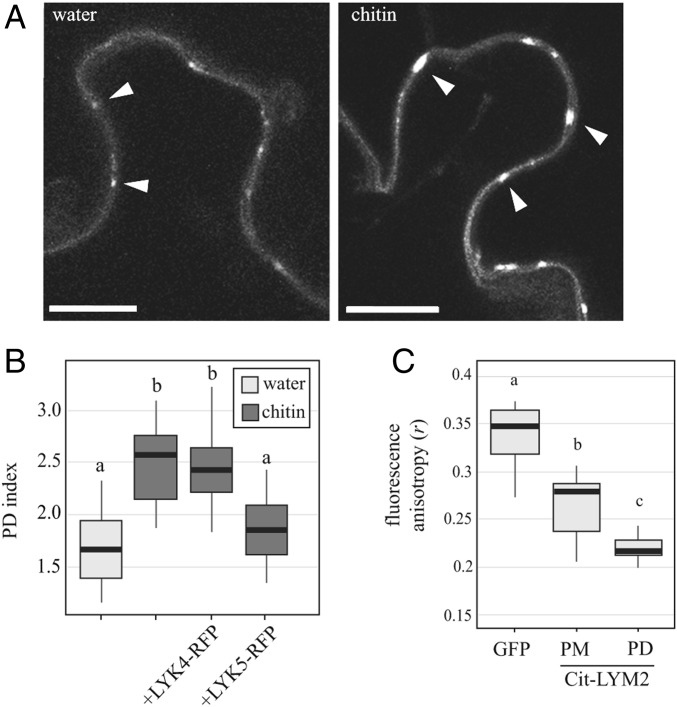
LYM2 accumulates at PD in response to chitin. (*A*) Single-plane confocal images of *N. benthamiana* tissue expressing Citrine-LYM2 before and after chitin treatment. *Left* shows Citrine-LYM2 in water-treated tissue, and *Right* shows Citrine-LYM2 30 min post chitin treatment. Arrowheads indicate example plasmodesmata. (Scale bars, 10 μm.) (*B*) Quantification of the PD index of Citrine-LYM2 in *N. benthamiana* after mock and chitin treatments. Number of images analyzed is ≥18. (*C*) Fluorescence anisotropy (*r*) of cytosolic GFP, PM located Citrine-LYM2, and plasmodesmata-located (PD) Citrine-LYM2. Number of images analyzed is ≥11. (*B* and *C*) Box plots: the line within the box marks the median, the box signifies the upper and lower quartiles, and the whiskers represent the minimum and maximum within 1.5 × interquartile range. Samples with the same letter code (a, b, or c) are not significantly different (*P *< 0.001).

Recent studies have identified that some receptors accumulate at plasmodesmata in response to stress (20, 21). Using live-cell imaging of Citrine-LYM2 in *N. benthamiana* leaves, we measured the plasmodesmal index (PD index) of fluorescence intensity (plasmodesmata:PM fluorescence; ref. 32) for Citrine-LYM2 in the absence and presence of chitin (Fig. 4*A*). The mean PD index of Citrine-LYM2 was significantly higher following 30 min of chitin treatment than it was following 30 min of water treatment. To determine if this process occurs also in *Arabidopsis*, we generated a complemented mutant line, i.e., *lym2 Arabidopsis* plants that express Citrine-LYM2 from its native promoter. In this line, chitin also induced an increase in the PD index relative to water treatment (*SI Appendix*, Fig. S4), suggesting that LYM2 accumulation is a genuine element of LYM2 signaling.

Using transient expression in *N. benthamiana*, we determined the effect of overexpressing LYK4 and LYK5 on LYM2 accumulation (Fig. 4*B*). A chitin-triggered increase in the PD index of LYM2 occurred when Citrine-LYM2 was coexpressed with LYK4-RFP but not LYK5-RFP, indicating that overexpression of LYK5 impairs LYM2 accumulation at plasmodesmata. This suggests that any complex formation with LYK4 does not impair mobility or function of LYM2 but that overexpression of LYK5 might saturate LYM2 and anchor it in the PM.

Accumulation of LYM2 at plasmodesmata suggests the possibility of higher-order complex formation in the plasmodesmal PM. To determine if LYM2 interacts with itself at plasmodesmata—as would be expected in such a complex—we performed fluorescence anisotropy measurements of Citrine-LYM2 in chitin-treated *N. benthamiana* tissue. We observed that anisotropy (*r*) of Citrine-LYM2 is lower in the PM relative to freely rotating cytosolic GFP, inferring that homo-FRET and LYM2 association occurs in the PM (Fig. 4*C*). *r* is further reduced in plasmodesmata, suggesting higher-order interaction of Citrine-LYM2 at plasmodesmata, indicative of a signaling platform in the plasmodesmal PM.

### LYM2-Dependent Chitin-Triggered Plasmodesmata Closure Engages Distinct Calcium/Reactive Oxygen Species Regulatory Modules.

Reactive oxygen species (ROS) are produced during immunity and can induce plasmodesmata closure (15, 33). Thus, we hypothesized that ROS play a role in the regulation of plasmodesmata closure in response to chitin, downstream of LYM2. We verified that H_2_O_2_-induced plasmodesmata closure is detected by the bombardment method, observing a reduction of GFP movement from cell to cell in WT plants after H_2_O_2_ treatment (*SI Appendix*, Fig. S5). In this assay, *lym2-1* mutants were able to close their plasmodesmata in response to H_2_O_2_, demonstrating that any role for ROS signaling in chitin-triggered plasmodesmata closure occurs independently, or downstream, of LYM2. By measuring chitin-triggered ROS production in *N. benthamiana* leaf discs, we also determined that Citrine-LYM2, LYK4-RFP, or LYK5-RFP induces enhanced ROS production (*SI Appendix*, Fig. S5), indicating that each of these LysM proteins can trigger ROS signaling and is functional as a fusion protein.

**Fig. 5.**
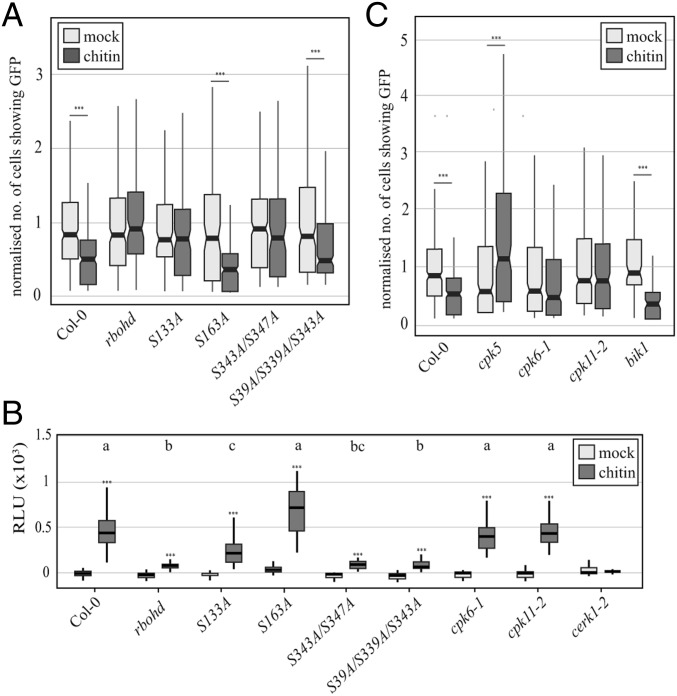
CPK-dependent phosphorylation of RBOHD is required for plasmodesmata closure in response to chitin. (*A*) Microprojectile bombardment into leaf tissue shows that *rbohd* mutants do not show a reduction in GFP movement to neighboring cells in response to chitin. RBOHD mutant variants RBOHD_S39A/S339A/S343A_ (S39A/S339A/S343A) and RBOHD_S163A_ (S163A) exhibit a reduction in movement of GFP to neighboring cells in response to chitin while the RBOHD phosphosite mutant variants RBOHD_S343A/S347A_ (S343A/S347A) and RBOHD_S133A_ (S133A) do not. (*B*) ROS produced by seedlings treated with chitin. Col-0, RBOHD_S133A_, RBOHD_S163A_, *cpk6-1* and *cpk11-2* seedlings all produce significantly more ROS than the *rbohd* mutant. Different letters (a, b, and c) indicate statistically significant groups when the chitin-triggered ROS are compared between genotypes (*P* < 0.05, analysis excluding *cerk1-2*); *** indicates significantly more ROS produced (*P* < 0.001) when chitin treatment is compared with water controls within genoptypes (all genotypes). Number of seedlings measured is ≥19. (*C*) Microprojectile bombardment into leaf tissue shows that *cpk6-1* and *cpk11-2* mutants do not show a reduction in GFP movement to neighboring cells in response to chitin. *cpk5* mutants show constitutively reduced movement that increases upon chitin treatment and *bik1* mutants behave like Col-0. (*A* and *C*) The number of cells showing GFP has been normalized to the mean of the mock data within genotypes. Box plots: the line within the box marks the median, the box signifies the upper and lower quartiles, and the minimum and maximum within 1.5 × interquartile range. Notches represent approximate 95% CIs. ***P < 0.001 (number of bombardment sites counted ≥ 89).

The rapid production of ROS in response to chitin is associated with the NADPH oxidase RBOHD (34). Bombardment assays showed that the *rbohd* mutant is not able to close its plasmodesmata in response to chitin treatment (Fig. 5*A* and *SI Appendix*, Fig. S7). This was supported by quantitative analysis of plasmodesmal callose that showed that *rbohd* mutants did not deposit callose at plasmodesmata in response to chitin (*SI Appendix*, Fig. S6). Thus, ROS produced by RBOHD are a critical component of the chitin-triggered signaling cascade that induces plasmodesmata closure.

In *Arabidopsis*, RBOHD is regulated by phosphorylation by several protein kinases (34–36). Receptor-like cytoplasmic kinases (RLCKs) are essential for the ROS burst triggered by CERK1 (34, 37). The RLCK BOTRYTIS-INDUCED KINASE 1 (BIK1) and CPKs both phosphorylate RBOHD in response to chitin: BIK1 phosphorylates Ser39, Ser339, Ser343, and Ser347, while CPKs phosphorylate Ser133, Ser148, Ser163, and Ser347 (34, 35). To test the dependence of chitin-triggered plasmodesmata closure on different phosphorylation sites within RBOHD, we investigated a range of phospho-null mutant variants (mutation of serine to alanine) of RBOHD in the *rbohd* mutant background: RBOHD_S39A/S339A/S343A_, RBOHD_S343A/S347A_, RBOHD_S133A_, and RBOHD_S163A_. RBOHD_S39A/S339A/S343A_ and RBOHD_S163A_ can close their plasmodesmata in response to chitin like wild-type Col-0 plants (Fig. 5*A* and *SI Appendix*, Fig. S7). By contrast, RBOHD_S343A/S347A_ and RBOHD_S133A_ cannot close their plasmodesmata in response to chitin. These data implicate Ser347 and Ser133 in chitin-triggered plasmodesmal closure.

To investigate the specificity of these phosphorylation sites to plasmodesmal responses, we further established the potential for these phospho-null mutant variants to produce a CERK1-mediated ROS burst in response to chitin. Relative to the *rbohd* mutant, Col-0, RBOHD_S133A_, and RBOHD_S163A_ produced significantly more chitin-triggered ROS, while neither RBOHD_S39A/S339A/S343A_ or RBOHD_S343A/S347A_ showed a difference in ROS production relative to *rbohd* (Fig. 5*B*). This demonstrates that the CERK1-mediated ROS production and the LYM2-mediated plasmodesmal response are specifically dependent on Ser39/Ser339/Ser343 and Ser133, respectively, implicating different phosphorylation signatures of RBOHD in distinct chitin signaling cascades.

### Calcium-Dependent Protein Kinases Mediate Chitin-Triggered Plasmodesmal Closure.

Both Ser347 and Ser133 have been shown to be phosphorylated by CPKs, and CPK5, -6, and -11 have each been described to phosphorylate RBOHD in vitro (34, 38). However, it is unclear whether any of these CPKs play a role in plasmodesmal closure. Notably, bombardment assays showed that *cpk6-1* and *cpk11-2* mutants were unable to close their plasmodesmata in response to chitin (Fig. 5*C* and *SI Appendix*, Fig. S7), while the plasmodesmata of the *cpk5* mutant opened in response to chitin (Fig. 5*C* and *SI Appendix*, Fig. S7). By contrast, *bik1* mutant plants showed a normal plasmodesmal response to chitin. Demonstrating specificity to plasmodesmal chitin responses, *cpk6-1* and *cpk11-2* mutants produced similar levels of chitin-triggered ROS to Col-0 plants (Fig. 5*B*). These data therefore identify CPK6 and CPK11 as critical components of the plasmodesmatal response to chitin and further illustrate the independence of the CERK1- and LYM2-initiated signaling pathways.

## Discussion

Receptor activation and signaling depend on the membrane environment in which the receptor resides; activation and signaling are frequently separated in a distinct membrane domain. Here, we have characterized how one ligand can activate different signaling cascades in different microdomains of the PM. Our data have identified that while chitin is perceived and signals via a CERK1-dependent cascade in the PM, chitin signaling in the plasmodesmal PM occurs via a LYM2/LYK4/LYK5-dependent cascade to produce a specific, localized response. In the plasmodesmal PM, both the receptors deployed and the downstream signaling cascade show specificity to their subcellular context and enable independent signaling in this microdomain.

Specificity in ligand perception and signaling to different cellular compartments is a feature of innate immune signaling in animal cells. For example, flagellin can be perceived extracellularly by the PM-anchored TOLL-LIKE RECEPTOR 5 (TLR5) (6) and in the cytosol by the soluble NOD-LIKE RECEPTOR FAMILY CARD DOMAIN CONTAINING 4 (NLRC4) (39, 40). At the response level, the LPS receptor TLR4 triggers signaling from the PM but also subsequently initiates a secondary signaling cascade from endosomes following internalization (7). Our findings here show that plant cells can combine both receptor and signaling specialization within a continuous membrane to fully integrate the perception of an immune signal.

Our investigation into the mechanisms of chitin signaling in the plasmodesmal PM identified that the plasmodesmata-resident GPI-anchored protein LYM2 accumulates at plasmodesmata in response to chitin. LYM2 also exhibits greater homo-FRET in the plasmodesmal PM than in the PM, indicating it oligomerizes or clusters there, possibly to form a signaling platform. It is well established that many forms of immune signaling occur via the formation of supramolecular complexes such as signalosomes and inflammasomes that involve the oligomerization of cytosolic NLR receptors and downstream signaling components. Membrane-anchored receptors also form multicomponent signaling complexes (41–43) and, in some cases, higher-order clusters (44, 45) for signaling. Like LYM2, several RKs were recently shown to accumulate at the plasmodesmal PM in response to salt stress (20, 21), suggesting that the plasmodesmal PM might commonly execute signaling via transient recruitment and concentration of machinery. Whether specific signaling cascades coexist in the plasmodesmal PM and ultimately induce the same response—as seen for LYM2 in chitin signaling and CML41 in flg22 signaling (16, 22)—requires further investigation of the plasmodesmal PM signaling cascade.

We found that, in addition to LYM2, the RKs LYK4 and LYK5 are also critical for plasmodesmal PM chitin signaling. For PM chitin signaling, a CERK1-LYK4-LYK5 tripartite complex has been suggested (albeit not biochemically demonstrated) (46). However, we did not find evidence to support a similar LYM2-LYK4-LYK5 complex in the plasmodesmal PM: we could not identify LYK5 in purified plasmodesmal extracts (Fig. 2*A*) or identify any interaction between LYK4 and LYK5 at plasmodesmata (Fig. 3*C*). We further observed that overexpression of LYK5 impaired LYM2 accumulation in the plasmodesmal PM (Fig. 3*B*), suggesting the LYM2-LYK5 association occurs in the PM. By contrast, LYK4 was detected in purified plasmodesmata extracts (Fig. 2*A*) and did not impair LYM2 accumulation at the plasmodesmal PM (Fig. 3*B*), thus supporting a model in which LYK4 and LYM2 are resident in the plasmodesmal PM and form a signaling complex.

With respect to the role of LYK5, our data demonstrate that LYK5 can associate with both LYM2 and LYK4 in the PM (Figs. 2*B* and 3*A*) and that a pool of LYK4 dissociates from LYK5 in response to chitin (Fig. 3*B*). Thus, it is possible that complex conformations are dynamic in a signaling context. We also observed that while LYK5 is not essential for the association between LYM2 and LYK4, it is essential for modification of the LYM2-associated pool of LYK4 (Fig. 2*C*). These data support the possibility that PM-resident LYK5 is critical for LYK4 function upstream of plasmodesmal signaling. Whether LYK5 association with LYK4 occurs in a tripartite LYM2-LYK4-LYK5 complex in the PM, or by a simpler bipartite LYK4-LYK5 complex, cannot be concluded without further biochemical analysis of the complex.

We identified that downstream of these receptors, chitin-triggered plasmodesmata closure is dependent on specific phosphorylated serines of RBOHD that are different from those required for the CERK1-mediated ROS burst (Fig. 5*B*). The serines critical for plasmodesmal responses are targeted by CPKs, while those targeted by the RLCK BIK1 are not required. Our experiments indicate that both CPK6 and CPK11 are positive regulators of plasmodesmal chitin responses (Fig. 5*C*), suggesting there is specificity of CPK function within this signaling cascade. CPK5 appears to oppositely regulate plasmodesmal chitin responses; the *cpk5* mutant exhibits reduced cell-to-cell movement of GFP in control conditions (*SI Appendix*, Fig. S7), suggesting that it functions in plasmodesmal regulation beyond the chitin-response context. It is possible that plasmodesmal signaling is dependent only on CPK-mediated activation of RBOHD or alternatively that other, uncharacterized residues targeted by RLCKs might also function in plasmodesmal signaling.

Given the complexity of factors that influence plasmodesmal permeability, we have given greater weight to comparisons within genotypes to identify a capacity for response than to comparisons of the magnitude of cell-to-cell movement of GFP observed between genotypes. However, examination of the raw data (*SI Appendix*, Figs. S2 and S7) suggests that plasmodesmata might be sensitive to changes in CPK and RBOHD function in mock conditions. Comparing genotypes, our data suggest the possibility that CPK5, CPK11, and phosphorylation of Ser133 and Ser163 of RBOHD negatively regulate plasmodesmal closure in mock conditions. It is possible that different CPKs and phosphorylation events act specifically as positive or negative regulators of NADPH oxidase activity or that different modes of NADPH oxidase activity convey different signals. However, independent of the mechanism, the data suggest that calcium and ROS signaling tune plasmodesmal responses across different conditions. This mechanism of plasmodesmal regulation, the specificity of the roles played by different CPKs, and the range of stimuli to which it is relevant require further investigation.

**Fig. 6.**
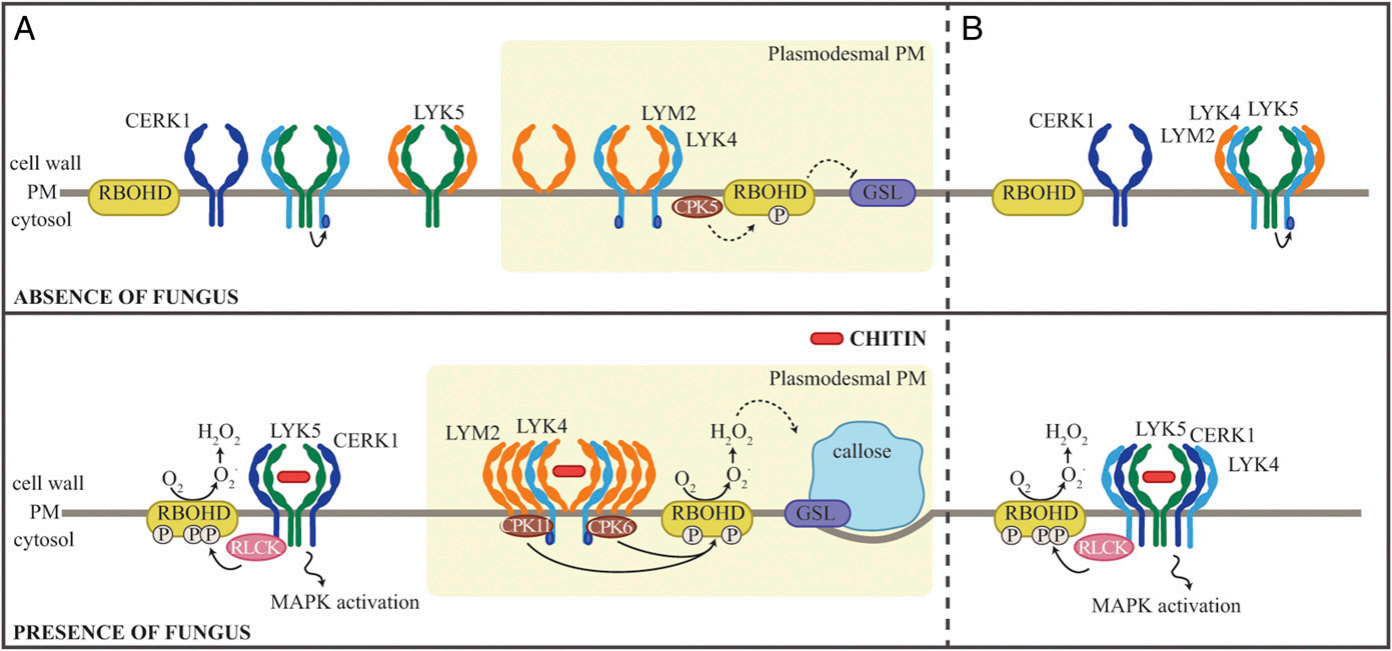
Possible mechanisms for LYM2-mediated chitin signaling in the plasmodesmal PM. This cartoon illustrates two possibilities for some key elements of LysM protein chitin signaling in the PM and plasmodesmal PM. *Top* represents the relevant associations and localizations we have identified under mock conditions (absence of fungus). Here, LYK5 (green) interacts with LYK4 (light blue), and LYM2 (orange) in the PM and LYK5 mediates modification of a pool of LYK4. This could occur via a population of bipartite complexes (*A*) or a tripartite LYM2-LYK4-LYK5 complex (*B*) in the PM. CPK5 negatively regulates callose synthesis in the plasmodesmal PM via a specific phosphorylation pattern (P) (white) of RBOHD. In response to chitin (*Lower*, presence of fungus), a pool of LYK4 and LYM2 dissociate from LYK5. LYK5 associates with CERK1 (dark blue) (*A*) or both CERK1 and LYK4 (*B*) to mediate signaling at the PM, and LYM2 accumulates at plasmodesmata, where it forms a higher-order complex or a signaling platform. This complex recruits LYK4 and CPK6 and -11 (brown) to phosphorylate (P) (white) RBOHD (yellow) at Ser133 and Ser347 and induces callose (blue) synthesis via a glucan synthase-like enzyme (GSL) (purple) to close PD. The PM LYK5-containing complex signals, in part, via RLCKs that phosphorylate (P) (white) RBOHD (yellow) at Ser39, Ser339, and Ser343 (P) (white). While not represented here, RLCKs might constitutively associate with LysM receptor complexes in the PM as for LRR-RKs (47).

Based on existing data, there are different possible models of the integration of chitin signals in the PM and the plasmodesmal PM. We present two possibilities here (Fig. 6). We suggest that both LYK4 and LYM2 associate with LYK5 at the PM and that, for LYK4, this mediates function-critical modification(s). This could occur via a mixed population of bipartite complexes (Fig. 6*A*) or a LYM2-LYK4-LYK5 tripartite complex (Fig. 6*B*) in the PM. Chitin perception triggers formation of a higher-order LYM2 complex, or signaling platform, in the plasmodesmal PM that recruits LYK4 and possibly CPK6 and CPK11. These CPKs phosphorylate Ser133 and Ser347 of RBOHD to produce ROS and induce localized callose synthesis, ultimately leading to plasmodesmata closure. CPK5 negatively regulates chitin-triggered plasmodesmata closure by phosphorylation of RBOHD specifically in the steady state such that it inhibits callose synthesis. Combining our data, and that of other studies, it is clear some interactions between LysM receptors are chitin-independent. Thus, it seems likely that the dynamics and associations that define LysM signaling have complexity and significance beyond simple ligand-dependent associations.

Our data identify that plasmodesmal chitin signaling involves specificity in both receptor identity and RBOHD regulatory modules. It remains to be determined if different mechanisms of activation of RBOHD result in different ROS outputs. However, the specificity in phosphorylation signatures observed, the lack of evidence of cross-talk between ROS produced by CERK1 and LYM2 (22), and the proximity of plasmodesmal PM and PM chitin-triggered signaling cascades all suggest that ROS signaling is highly localized in immune responses. The requirement for specific signaling at the plasmodesmal PM further suggests that cell-to-cell connectivity must be regulated independently of other immune outputs, raising questions of whether there is a critical requirement for cells to balance connectivity and resource exchange with a protective mechanism imposed by isolation.

Importantly, we have demonstrated that the plasmodesmal PM integrates signals independently of the PM and that plant cells exploit receptor and signaling specificity to execute localized responses. Membrane domain specialization enables independent control of plasmodesmata in immune signaling. As plasmodesmata are regulated by a range of signals, it seems likely that this also occurs in other contexts. Indeed, as there are a variety of membrane domains across the PM, it is possible that specific signaling underpins many receptor-triggered processes in plants.

## Material and Methods

Extended methods are available in *SI Appendix*.

### Plant Materials.

*Arabidopsis* Columbia (Col-0) is wild type. The mutant lines used in this study are *rbohd* (48), *lym2-1* (22), *cerk1-2* (49), *lyk3* (27), *lyk4* (27), *lyk5-2* (23), *bik1* (50), *cpk5* (35), *cpk11*-*2* (51), and *cpk6-1* (52). RBOHD variants RBOHD_S343A/S347A_, RBOHD_S39A/S339A/S343A_, RBOHD_S133A_, and RBOHD_S163A_ are mutant variants of RBOHD transformed in to the *rbohd* mutant background (34). *lym2/LYM2pro::Citrine-LYM2* plants were made by transformation of *lym2-1* with the *LYM2pro::Citrine-LYM2* construct.

### Microprojectile Bombardment Assays.

Microprojectile bombardment assays were performed as described (22). Bombardment sites were assessed 16 h after bombardment by confocal or epifluorescence microscopy with a 25× water-dipping objective (HCX IRAPO; 25.0 × 0.95 water). The number of cells showing GFP was counted for each bombardment site.

### Plasmodesmal Callose Staining and Quantification.

Plasmodesmal callose staining and quantification were performed as described (16). Three z-stacks from three areas per leaf were imaged. This was replicated for 5 to 12 leaves per genotype and treatment. Aniline blue-stained plasmodesmal callose was quantified using the automated image analysis pipeline “find plasmodesmata” (16) (https://github.com/JIC-CSB/find-plasmodesmata). All annotated images were sanity-checked prior to inclusion in Dataset S1.

### PD Index.

Leaves of *N. benthamiana* transiently expressing the constructs of interest, or *lym2*/*pLYM2::Citrine-LYM2 Arabidopsis* plants, were infiltrated with chitin (500 µg/mL) or water (mock conditions) and stained with 0.1% aniline blue. The abaxial side of the leaf was imaged using a 63× water-immersion objective lens (C-APOCHROMAT; 63×/1.2 water) with a confocal microscope. PD index was determined by measuring the intensity values of Citrine-LYM2 in ROIs that represent plasmodesmata and neighboring PM regions with ImageJ.

### Fluorescence Anisotropy.

Leaves of *N. benthamiana* transiently expressing Citrine-LYM2 and cytosolic GFP were imaged with a Leica SP8 confocal microscope. Citrine was excited with a pulsed white light laser (488 nm), and emitted light was separated into parallel and perpendicular polarizations and detected by external single photon avalanche diodes with 500- to 550-nm filters. A series of 20 frames was merged and analyzed using PicoQuant SymPhoTime 64. Anisoptropy (*r*) was calculated by

$$r=\frac{I_{∥}-GI_{⊥}}{\left(2-3L_{1}\right)I_{⊥}\;+\left(1-3L_{2}\right)I_{∥}},$$

where *G* = 0.481, *L*_1_ = 0.013, and *L*_2_ = 0.037. ROIs were defined that correlated to plasmodesmata and the PM for Citrine-LYM2 images or the cytosol for GFP images.

### FRET-FLIM Analysis.

Leaves of *N. benthamiana* transiently expressing the constructs of interest were used 30 min after infiltration with chitin (500 µg/mL) or water (mock conditions) for FRET-FLIM. The abaxial side of the leaf samples was imaged using a 63×/1.2 water-immersion objective lens (Leica C-APOCHROMAT; 63×/1.2 water). FLIM experiments were performed using a Leica TCS SP8X confocal microscope equipped with time-correlated single-photon counting electronics (PicoHarp 300), photon-sensitive detectors (HyD SMD detector), and a pulsed white light laser (470 to 670 nm). GFP was excited at 488 nm (40 MHz) and collected between 509 to 530 nm. Data analysis is described in full in *SI Appendix*.

### FRAP Analysis.

FRAP was performed using a Leica TCS SP8X CLSM with a 63×/1.20 water-immersion objective (Leica HC PL APO CS2; 63×/1.20). ROIs were defined for plasmodesmata-localized Citrine-LYM2 and for PM-localized Citrine-LYM2, LYK4-RFP, and LYK5-RFP. Fluorescence recovery was measured in ROIs 60 s postbleach. Data analysis is described in full in *SI Appendix*.

### Co-IP.

*Arabidopsis* protoplasts and *N. bethamiana* leaves were used for immunprecipitation experiments. Proteins were extracted in IP buffer containing 50 mM tris(hydroxymethyl)aminomethane∙HCl (pH 7.5), 150 mM NaCl, 5 mM dithiothreitol, protease inhibitor mixture (Sigma) 1:100, phosphatase inhibitor (Sigma) 1:200, 1 mM phenylmethanesulfonyl fluoride, 0.5% IPEGAL CA-630 (Sigma), 1 mM ethylenediaminetetraacetic acid, 1 mM Na_2_MoO_4_ × 2H_2_O, 1 mM NaF, and 1.5 mM activated Na_3_VO_4_. For co-IP, GFP-Trap agarose or magnetic beads (ChromoTek) were incubated with the protein samples for 2 h at 4 °C with gentle agitation. Beads were washed at least three times with IP buffer and proteins released by heating to 95 °C in Laemmli buffer (2×).

### Plasmodesmata Extraction.

The plasmodesmal purification method from *Arabidopsis* suspension cultures cells (53) was modified for use with mature *N. benthamiana* leaf tissue. The modified method is described in detail in *SI Appendix*.

### ROS Burst Measurement.

For measurements of chitin-triggered ROS production seedlings, plants were grown in 96-well plates in half-strength Murashige and Skoog medium supplemented with 0.5% sucrose for 11 to 12 d under 16-h light/10-h dark conditions. Plants were incubated overnight in water and assayed in 20 µg/mL HRP and 6 µM L-012 in water, with or without 500 µg/mL chitin. Chemiluminescence was recorded using a Varioskan Flash (Thermo Fisher), and luminescence was emitted in the first 25 min after elicitation was integrated, corrected for background luminescence, and used for subsequent analysis.

### Statistical Analyses.

Statistical analyses were performed using Genstat Version 18 or R Version 3.5.1. Unless otherwise indicated, statistical significance was concluded when the *P* value was less than 0.05; *n* values are listed in *SI Appendix*, Table S3. Details for the design and statistical analysis of each experiment are detailed in *SI Appendix*, *Extended Methods*.

### Data Availability.

All data measurements used in this paper are available in Dataset S1. R scripts are available in GitHub (54).

## Supplementary Material

Supplementary File

Supplementary File
