# Supplementary material for: Chitin perception in plasmodesmata characterizes submembrane immune-signaling specificity in plants
Source: Proc Natl Acad Sci U S A. 2020 Apr 13;117(17):9621–9. doi: 10.1073/pnas.1907799117 (PMC7196898; doi:10.1073/pnas.1907799117)
Supplement: Supplementary File [file pnas.1907799117.sapp.pdf]

Supporting Information

Expanded Material and Methods

Plant materials

Arabidopsis plants were grown on soil in short days conditions with 10 h light at 22 °C. *N. benthamiana* plants were grown on soil with 16 h light at 23 °C. *Arabidopsis* Columbia (Col-0) is wild-type. The mutant lines used in this study are referenced in the main text: *rbohd*, *lym2-1* (SAIL_343_B03), *cerk1-2* (GABI_096F09), *lyk3* (SALK_140374), *lyk4* (WiscDsLox297300_01C), *lyk5-2* (SALK_131911.31.20.x), *bik1* (SALK_005291C), *cpk5* (SAIL_657C06), *cpk11-2* (SALK_054495) and *cpk6-1* (SALK_093308). RBOHD variants $RBOHD_{S343A/S347A}$, $RBOHD_{S39A/S339A/S343A}$, $RBOHD_{S133A}$ and $RBOHD_{S163A}$ are mutant variants of RBOHD transformed in to the *rbohd* mutant background. Complemented *lym2/LYM2pro::Citrine-LYM2* plants were made by transformation of *lym2-1* with the *LYM2pro::Citrine-LYM2* construct described below.

DNA Constructs

35S::Citrine-LYM2 is as previously described (1). *LYK4* and *LYK5* coding sequences were PCR amplified from Col-0 cDNA and recombined into pB7FWG2.0, pB7RWG2.0 and pGWB14 to make C-terminally-tagged fusions with GFP, RFP or HA. The BRI1-RFP fusion is as described (2). For generation of $RBOHD_{S133A}$ and $RBOHD_{S163A}$ we used site-directed mutagenesis: DNA fragments were amplified by PCR and cloned into the pBin19g vector. To construct a *Citrine-LYM2* expression module with native regulatory elements, the promoter (1.4 kbp upstream of the start codon) and terminator (0.4 kbp downstream of the stop codon) of *LYM2* were PCR amplified and cloned in fusion with *Citrine-LYM2*. The expression module was ligated into the binary plasmid pICSL4723.

Transient expression in *Nicotiana benthamiana*

Agrobacterium tumefaciens GV3101 carrying the desired construct was cultured overnight and resuspended in 0.01 M 2-(N-morpholino)ethanesulfonic acid (MES) pH 5.6, 0.01 M $MgCl_2$, 0.01 M acetosyringone. Each *Agrobacterium* strain carrying the desired construct was mixed with an *Agrobacterium* strain carrying the P19 silencing suppressor and syringe-infiltrated into leaves of 4-week-old *Nicotiana benthamiana* plants. Material for experiments was harvested two days post-infiltration.

Semi-quantitative RT-PCR analysis

RNA was extracted from leaves of 5-week-old *Arabidopsis* plants using RNeasy Mini extraction kit (Qiagen) and treated with Turbo DNA-free kit (Ambion) before cDNA synthesis using Reverse Transcriptase (Promega) according to the manufacturer's instructions. Semi-quantitative RT-PCR was performed on the cDNA samples with primers listed in Table S2.

Microprojectile bombardment assays

Microprojectile bombardment assays were performed as described (1). Expanded leaves of five- to six-week-old *Arabidopsis* plants were bombarded with 1 nm gold particles (BioRad) coated with pB7WG2.0-GFP and pB7WG2.0-RFP_{ER}, using a Biolostic PDS-1000/He particle delivery system (BioRad). Bombarded leaves were infiltrated with 500 µg/mL chitin (mixture of chitin oligosaccharides, NaCoSy), 150 mM H_2O_2, or water 2 h post bombardment. Bombardment sites were assessed 16 h after bombardment by confocal (Leica SP5 or SP8) or epifluorescence microscopy (Leica DM6000) with a 25× water dipping objective (HCX IRAPO 25.0× 0.95 water). The number of cells showing GFP was counted for each bombardment site (marked by RFP_{ER}).

Protoplast preparation and transfection

Protoplasts were extracted and transfected as described previously (3). True leaves from 4- to 5-week-old *Arabidopsis* plants were cut in 1 mm leaf strips using a razor blade. Leaf strips were digested in the dark for 3h in 20 mM MES (Sigma) pH 5.7, 1.5 % (w/v) cellulase R10 (Yakult Pharmaceuticals, Japan), 0.4 % (w/v) macerozyme R10 (Yakult Pharmaceuticals, Japan), 0.4 M mannitol, 20 mM KCl, 10 mM $CaCl_2$ and 0.1 % bovine serum albumin (BSA). Protoplasts were diluted with an equal volume of W5 solution (2 mM MES pH 5.7, 154 mM NaCl, 125 mM $CaCl_2$, 5 mM KCl) and filtered with a 75 µm nylon mesh. Protoplasts were pelleted at 150 x *g* for 1 min and re-suspended at $2x10^5$ mL^{-1} in W5 solution. Protoplasts were kept on ice for 30 min, pelleted at 150 x *g* and re-suspended at $2x10^5$ mL^{-1} in 4 mM MES pH 5.7, 0.4 M mannitol and 15 mM $MgCl_2$ at room temperature. For transfection, 10 µg of plasmid DNA was mixed gently with 100 µL of protoplasts and 110 µL PEG/calcium solution (20 % (w/v) PEG4000, 0.2 M mannitol and 100 mM $CaCl_2$). The transfection mixture was incubated at room temperature for 5 min, diluted with W5 solution and centrifuged at 100 x *g* for 2 min. Protoplasts were then re-suspended in 4 mM MES pH 5.7,

0.5 M mannitol, 20 mM KCl and incubated at room temperature overnight. Transfected protoplasts were collected by centrifugation (100 x *g* for 2 min).

Microscopy

Confocal microscopy was performed on a Leica SP5, Leica SP8, or ZEISS LSM800 with a 25× water-dipping lens (HCX IRAPO 25.0 × 0.95 water), a 40× oil immersion lens (HCX PLAPO CS 40.0× 1.25 OIL), a 63× oil immersion lens (Plan-APOCHROMAT 63×/1.4 oil) or a 63×/1.2 water immersion objective lens (C-APOCHROMAT 63×/1.2 water). Citrine was excited with a 488 nm or 514 nm argon laser and collected at 525–560 nm. mRFP and mCherry were excited with a 561 nm DPSS laser and collected at 600-640 nm, GFP was excited with a 488 nm argon laser and collected at 505-530 nm, aniline blue was excited with a 405 nm UV laser and collected at 430-550 nm.

Plasmodesmal callose staining and quantification

Plasmodesmal callose staining and quantification was performed as described (4). The 8th leaf of five- to six-week-old *Arabidopsis* plants was infiltrated with water or chitin. One hour after treatment, the leaf tissue was infiltrated with 0.01 % aniline blue in PBS buffer (pH 7.4). Callose deposits were imaged from the abaxial side of the leaf using a 63× oil immersion lens (Plan-APOCHROMAT 63×/1.4 oil) with a Leica SP5 confocal microscope. Aniline blue was excited with a 405 nm UV laser and collected at 430-550 nm. Three z-stacks from three areas per leaf were imaged. This was replicated for 5-12 leaves per genotype and treatment. Aniline blue stained plasmodesmal callose was quantified using the automated image analysis pipeline “find plasmodesmata” (4) (https://github.com/JIC-CSB/find-plasmodesmata). Three-dimensional segmentation was carried out by initially thresholding the input image (experimentally adjusted). The resulting binary image was then segmented by connected component analysis and filtered by the number of voxels in each component. Objects <2 voxels (noise) and objects >100 voxels (callose accumulated in stomata) were removed. All annotated images were sanity checked prior to inclusion of data in the final set.

PD index (plasmodesmata/PM fluorescence intensity ratio)

Leaves of *N. benthamiana* transiently expressing the constructs of interest, or *lym2*/*pLYM2::Citrine-LYM2 Arabidopsis* plants, were infiltrated with chitin (500 µg/mL) or water (mock conditions) and stained with 0.1% aniline blue for PD index determination. The abaxial side of the leaf samples was imaged using a 63×/1.2 water immersion objective lens

(C-APOCHROMAT 63×/1.2 water) with either a Leica SP8 or Zeiss LSM800 confocal microscope. PD index was determined by measuring the intensity values of Citrine-LYM2 in ROIs that represent plasmodesmata and neighbouring PM regions with ImageJ.

Fluorescence Anisotropy

Leaves of *N. benthamiana* transiently expressing Citrine-LYM2 and cytosolic GFP were imaged with a Leica SP8 confocal microscope. Citrine was excited with a pulsed white light laser (488 nm) and emitted light was separated in to parallel and perpendicular polarizations and detected by external SPADs with 500-550 nm filters. A series of 20 frames were merged and analysed using PicoQuant SymPhoTime 64. Anisoptropy (*r*) was calculated by

$$r = \frac{I_{\parallel} - GI_{\perp}}{(2 - 3L_1)I_{\perp} + (1 - 3L_2)I_{\parallel}}$$

where G=0.481, L_1=0.013 and L_2=0.037. ROIs were defined that correlated to plasmodesmata and the PM for Citrine-LYM2 images or the cytosol for GFP images.

FRET-FLIM analysis

Leaves of *N. benthamiana* transiently expressing the constructs of interest were used 30 min after infiltration with chitin (500 µg/mL) or water (mock conditions) for FRET-FLIM. The abaxial side of the leaf samples was imaged using a 63x/1.2 water immersion objective lens (Leica C-APOCHROMAT 63×/1.2 water). FLIM experiments were performed using a Leica TCS SP8X confocal microscope equipped with TCSPC (time correlated single photon counting) electronics (PicoHarp 300), photon sensitive detectors (HyD SMD detector), a pulsed laser (white light laser, WLL) with a range from 470 to 670 nm. The WLL at 488 nm was used to excite GFP at 488nm. Laser power was kept low (0.5-3%) to avoid any bleaching of the samples. GFP emission was collected between 509-530 nm. The repetition rate was set up to 40 Mhz. Additional 488 nm and 561 nm notch filters were used to reduce interference reflected light. The instrument response function (IRF) was measured using erythrosine B as described (5). The FLIM data sets were recorded using the Leica LASX FLIM wizard linked to the PicoQuant SymPhoTime 64 software. The FLIM data sets were acquired by scanning each image until a suitable number of photon counts per pixel (minimum 1000) was reached. For the acquisition, the image size was set to 250 × 50 pixels, allowing a pixel dwell time of 19 µs. The laser power was adjusted to reach a maximum count of 2000 kcounts per second. Each acquisition was stopped after 40 frames. Data were analysed by obtaining excited-state

lifetime values of a region of interest (PM or plasmodesmata). Calculations were performed using the PicoQuant SymPhoTime 64 software instructions for FLIM-FRET-Calculation for Multi-Exponential Donors, using a two-exponential decay for GFP. The lifetimes were initially estimated by fitting the data using the Monte Carlo method and then by fitting the data using the Maximum Likely Hood Estimation (MLE). The amplitude weighted average donor lifetime with model parameter n=2 was used to calculate the average FRET-efficiency. FRET efficiency (E) was calculated by comparing the lifetime of the donor in the presence τDA or absence τD of the acceptor according to the following formula: $E = 1 - \frac{\tau DA}{\tau D}$.

FRAP analysis

Leaves of *N. benthamiana* transiently expressing *Citrine-LYM2*, *LYK4-RFP* or *LYK5-RFP* were treated for 30 min with chitin (500 µg/mL) or water (mock). FRAP was performed using a Leica TCS SP8X CLSM with a 63x/1.20 water immersion objective (Leica HC PL APO CS2 63×/1.20). Citrine was excited at 514 nm and emissions detected between 527-550 nm. RFP was excited at 561 nm and emissions detected between 567-617 nm. ROIs were defined for plasmodesmata-localised Citrine-LYM2 and for PM-localised Citrine-LYM2, LYK4-RFP and LYK5-RFP. The FRAP protocol was: 30 iterations were imaged at 0.095 sec/frame (pre-bleach); 15 (Citrine) or 60 (RFP) iterations at 0.095 sec/frame (bleach); and 50 iterations at 0.095 sec/frame followed by 120 iterations every 0.5 s (post-bleach). For bleach iterations the laser power was set to 100% and the FRAP booster was used.

Intensity data were normalised to the mean intensity of the first five frames and corrected for bleaching induced by pre- and post-bleach iterations. For the latter we collected non-bleached image acquisition decay curves. These data were themselves normalised to mean intensity of the first five frames and were fitted by a LOESS regression. Thus, all experimental FRAP data was corrected as follows:

$$I_{corr} = I_{norm} + \left(100 - I_{decay}\right) \; [\%]$$

I_{corr} is the corrected intensity, I_{norm} is the normalised intensity, I_{decay} is the modelled intensity from the acquisition decay curve.

Data was further transformed to set the bleach baseline to zero: thus

$$I_{final} = \frac{I_{corr} - I_{bleach}}{I_{pre-bleach}} * 100 \; [\%]$$

I_{final} is the final intensity, I_{bleach} is the intensity at the last bleach interation, $I_{pre\text{-}bleach}$ is the mean of the pre-bleach values.

A LOESS curve was modelled to each dataset and each curve fit was sanity checked (6). The intensity value at 60 s post-bleach was used as an approximation of the relative mobile fraction, adapted from (7).

Co-immunoprecipitation

Proteins were extracted in immunoprecipitation (IP) buffer containing 50 mM Tris-HCl pH 7.5, 150 mM NaCl, 5mM dithiothreitol (DTT), protease inhibitor cocktail (Sigma) 1:100, phosphatase inhibitor (Sigma) 1:200, 1 mM phenylmethanesulfonyl fluoride (PMSF) and 0.5% IPEGAL® CA-630 (Sigma), 1 mM EDTA, 1 mM $Na_2MoO_4 \times 2H_2O$, 1 mM NaF, 1.5 mM activated Na_3VO_4. For co-immunoprecipitation, GFP-Trap agarose or magnetic beads (ChromoTek) were incubated with the protein samples for 2 h at 4 °C with gentle agitation. Beads were washed at least three times with IP buffer and proteins released by heating to 95 °C in Laemmli buffer (2×). Proteins were separated by SDS-PAGE and transferred to Immuno-blot® PVDF membrane. Proteins were detected with anti-GFP (Roche, 11 814 460 001), anti-HA (Sigma, H3663), anti-RFP-biotin (Abcam, ab34771) antibodies. We used anti-mouse-HRP (Sigma, A0168) and anti-rabbit–Alkaline Phosphatase (Sigma, A3687) for detection of the primary antibodies.

Plasmodesmata extraction

The plasmodesmal purification method from *Arabidopsis* suspension cultures cells (8) was modified for use with mature leaf tissue. Four expanded 5-week-old *N. benthamiana* leaves, transiently expressing the desired construct, were ground in liquid nitrogen to a fine powder. The powder was ultrasonicated in extraction buffer (50 mM Tris-HCl pH 7.5, 150 mM NaCl, 1× cOmplete™ ULTRA EDTA-free protease inhibitors (Roche), 1 mM PMSF, 1 % w/v PVP-40kDa (Sigma)), followed by high-pressure homogenisation (EmulsiFlex B15, Avestin) to produce the "Total" fraction. Triton-X100 (0.5 % v/v) was added to the resultant homogenate and the sample was centrifuged at 400 x *g* at 4 °C to collect a crude cell wall extraction. Cell walls were washed five times with extraction buffer, and once in cellulase buffer (20 mM MES-KOH pH 5.5, 100 mM NaCl, 1× cOmplete™ ULTRA EDTA-free protease inhibitors (Roche), 1 mM PMSF, 4.4 % w/v mannitol). Washed cell wall material was resuspended in an equal volume of cellulase buffer with 2 % (w/v) Cellulase R-10 (Yakult pharmaceuticals, Japan) and shaken at 37 °C for 1 h. Undigested cell wall material

was removed by centrifugation at 2500 x *g* at 4 °C. The supernatant was ultracentrifuged at 130,000 x *g* at 4 °C to collect plasmodesmal membranes. The membrane pellet was resuspended in 50 µL resuspension buffer (50 mM Tris-HCl pH 7.5, 150 mM NaCl, 5 mM DTT, 1× cOmplete™ ULTRA EDTA-free protease inhibitors (Roche), 1 mM PMSF, 0.2 % v/v IPEGAL® CA-630 (Sigma)) as a "PD" fraction. For extractions to detect H^+ ATPase 10 % glycerol was added to the extraction buffer and the cellulase buffer. Protein extracts were boiled in Laemmli buffer and separated by 10 % SDS-PAGE. Proteins were transferred to Immuno-blot® PVDF (0.2 µm, Bio-Rad) and the membrane was probed with primary-conjugated HRP antibodies against HA (ab173826, Abcam) and anti-H^+ ATPase (AS07 260, Agrisera). H^+ ATPase antibodies were detected with anti-rabbit-HRP (Sigma, A0545).

ROS burst measurement

For ROS assays in *N. benthamiana*, leaf discs (4 mm in diameter) were immersed in in 96-well white plates (Greiner bio-one) containing sterile water overnight in the dark. The next day, the water was replaced by a solution containing 20 µg/mL horse radish peroxidase (HRP; Sigma), 17 µg/mL luminol (Sigma), and chitin (NaCoSy, 500 µg/mL) or water (mock). Integrated luminescence was measured using a luminometer (Varioskan Flash) for 40 min. For measurements of chitin-triggered ROS production in RBOHD mutants, seedlings were grown in 96-well plates in half strength MS medium supplemented with 0.5 % sucrose for 11-12 d under 16-h light/10-h dark conditions. Plants were incubated overnight in water and assayed in 20 µg/mL HRP and 6 µM L-012 in water, with or without 500 µg/mL chitin. Chemiluminescence was recorded using a Varioskan Flash (Thermo Fisher) and luminescence emitted in the first 25 min after elicitation was integrated, corrected for background luminescence and used for subsequent analysis.

Statistical analyses

Statistical analyses were performed using Genstat® version 18 or R 3.5.1. Unless otherwise indicated, statistical significance was concluded when *p* value was less than 0.05.

Bombardment data: For bombardment experiments n is a single transformed cell. Data was pooled from bombardment events from 3 individual experiments (shots) each of which contained leaves from 3 individual plants. Unless otherwise indicated, data were analysed using a non-parametric resampling method (9). The absolute difference in medians of the observed data was calculated. This difference was compared to a null distribution of differences made of 10000 random permutations of the data, under the assumption that the

null hypothesis was true. The estimated *p* value was calculated as the number of pseudo-replicates which had an equal or greater absolute difference in medians plus one divided by 10001. *p* values were corrected for multiple comparisons using the Holm-Bonferroni method.

Callose quantification: For callose staining, mean intensity measurements are per image. 9 images were collected from a single leaf and 5-12 leaves were analysed for each treatment and genotype. Images were sanity checked before inclusion in the final data set. The normality of each data set was assessed by a Shapiro-Wilk test. When normally distributed a 2-tailed Student's t-test (assuming unequal variance) was applied to compare mock and chitin treated samples within genotypes. When the data was not normally distributed a non-parametric Mann-Whitney U test was used.

PD index: PD index values were averaged for each image images were collected from at least 3 independent leaves. For the *N. benthamiana* experiment, data was analysed by ANOVA with Tukey-Kramer post-hoc analysis for multiple comparisons. For the *Arabidopsis* experiment, data was tested using a 2-tailed Student's t-test, assuming unequal variance.

Fluorescence Anisotropy: *r* values per image represent measurements from multiple ROIs. Data was analysed by ANOVA with Tukey-Kramer post-hoc analysis for multiple comparisons.

FRET-FLIM: Data was analysed either by Student's t-test (2-tailed, assuming unequal variance), or by ANOVA with Tukey-Kramer post-hoc analysis for multiple comparisons.

FRAP: Data was transformed by LOESS regression analysis as outlined above and comparisons of estimated marginal means were performed in R 3.5.1 (R Core Team, 2018) using emmeans (10).

ROS assays: For *N. benthamiana* data, each data point represents a single leaf disc and analysis was by Mann-Whitney U test. For *Arabidopsis* seedlings, each data point represents a single seedling and differences within treatments among genotypes were tested using a Kruskal-Wallis test. A Dunn post-hoc test was subsequently used to test for pairwise differences (employing an FDR correction for multiple comparisons). Within genotype comparisons were made by Mann-Whitney U test.

Supplemental Figures and Tables

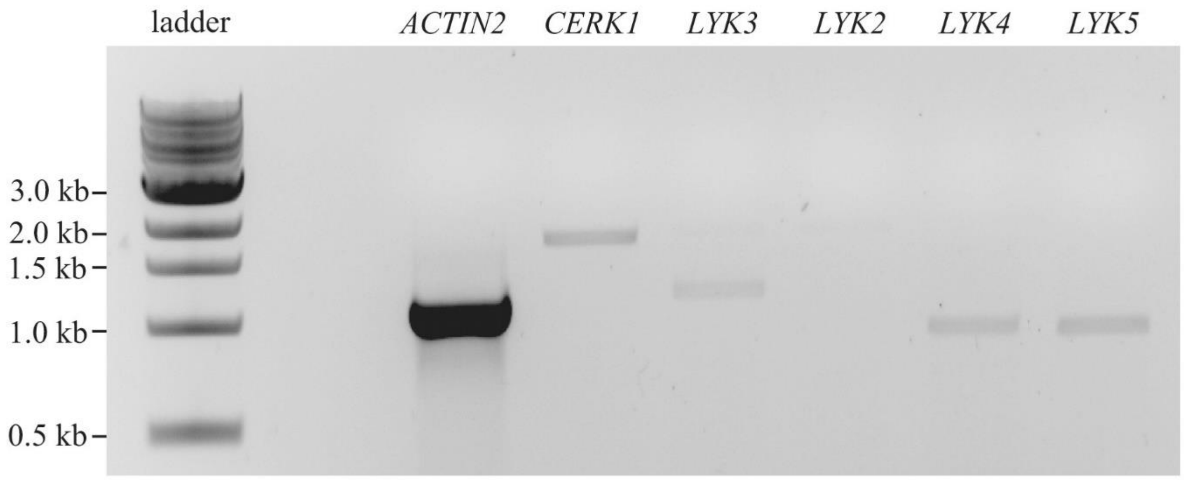

Figure S1. *LYK2* is not detected in mature leaves

Semi-quantitative transcript abundance of *LYSM-RKs* in 5- to 6-week-old *Arabidopsis* leaf tissue. Transcript abundance was detected by RT-PCR and *ACTIN2* was used as an internal control. The primers used for RT-PCR are listed in the supplemental data (Table S2). RT-PCR reactions with these primers give the following sizes: *ACTIN2*, 1131 bp; *CERK1*, 1854 bp; *LYK2*, 1960 bp; *LYK3*, 1275 bp; *LYK4*, 1083 bp; *LYK5*, 1082 bp.

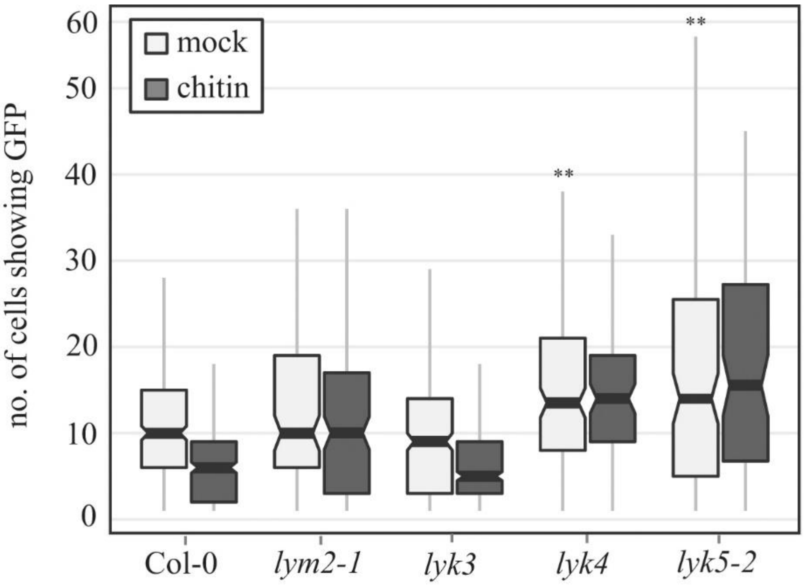

Figure S2. Raw, non-normalised data from bombardment experiments on *lym2-1* and *lyk* mutants.

Microprojectile bombardment data as described in Figure 1A. Analysis of non-normalised data shows that *lyk4* and *lyk5-2* exhibit a baseline increase in GFP flux from cell-to-cell. The data is summarised in box-plots in which the line within the box marks the median, the box signifies the upper and lower quartiles, the whiskers represent the minimum and maximum within 1.5 × interquartile range. Notches represent approximate 95 % confidence intervals. Number of bombardment sites (n) counted is indicated in Figure 1A. Asterisks indicate statistical significance compared to control conditions (***p*-value < 0.01).

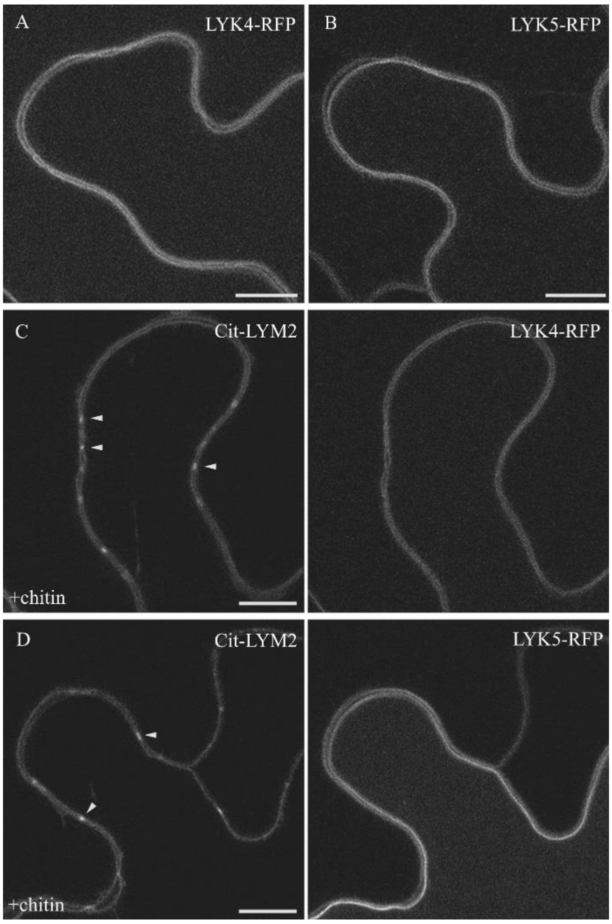

Figure S3. LYK4 and LYK5 localise to the PM.

LYK4-RFP (A) and LYK5-RFP (B) localise evenly across plasma membrane when transiently expressed in *N. benthamiana*. (C) and (D) show localisation of LYK4-RFP and LYK5-RFP respectively in tissue that is co-expressing Citrine-LYM2 (Cit-LYM2) and has been treated with chitin for 30 min. Arrowheads identify Citrine-LYM2 marked plasmodesmata. Scale bars are 10 µm.

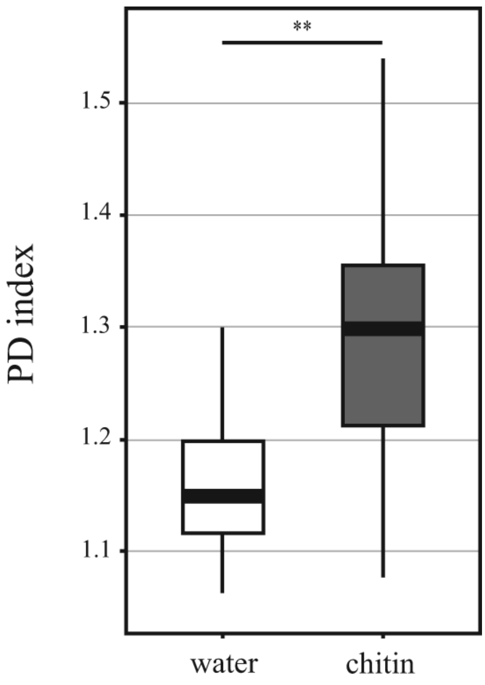

Figure S4. Chitin-induced PD index of *lym2/LYM2pro::Citrine-LYM2* plants.

Chitin triggers an increase in the PD index of *lym2/LYMpro::Citrine-LYM2*. Data represents images collected from leaves harvested in two independent experiments. Box plots represent data as follows: the line within the box marks the median, the box signifies the upper and lower quartiles, the whiskers represent the minimum and maximum within 1.5 × interquartile range. ***p*-value < 0.01, n values are indicated at the base of the graph.

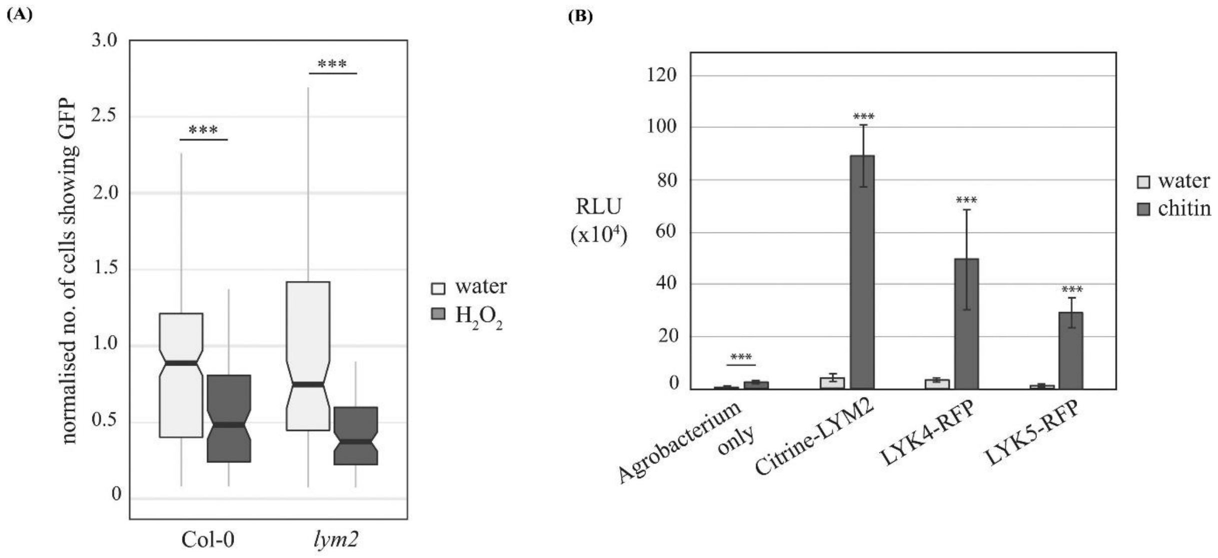

Figure S5. LYM2 acts upstream of ROS signalling in plasmodesmata closure

(A) Microprojectile bombardment into leaf tissue shows that H_2O_2 induces a reduction in GFP movement between cells in both Col-0 and *lym2-1* leaves. The number of cells showing GFP has been normalised to the mean of the mock data within genotypes. This data is summarised in box-plots in which the line within the box marks the median, the box signifies the upper and lower quartiles, the minimum and maximum within 1.5 × interquartile range. For water-treated samples, data is as presented in Fig 1. Data was analysed by a Mann-Whitney U-test for within genotype comparisons ($n \geq 85$, ***p-value < 0.001). (B) Total accumulation of ROS in chitin-treated *N. benthamiana* leaf discs transiently expressing Citrine-LYM2, LYK4-RFP or LYK5-RFP. Error bars are SEM and data was analysed by a Mann-Whitney U-test comparing the effect of chitin on Agrobacterium only control tissue and the magnitude of the chitin-induced ROS burst between the Agrobacterium only control and tissue expressing Citrine-LYM2, LYK4-RFP or LYK5-RFP ($n \geq 10$, ****p*-value < 0.001).

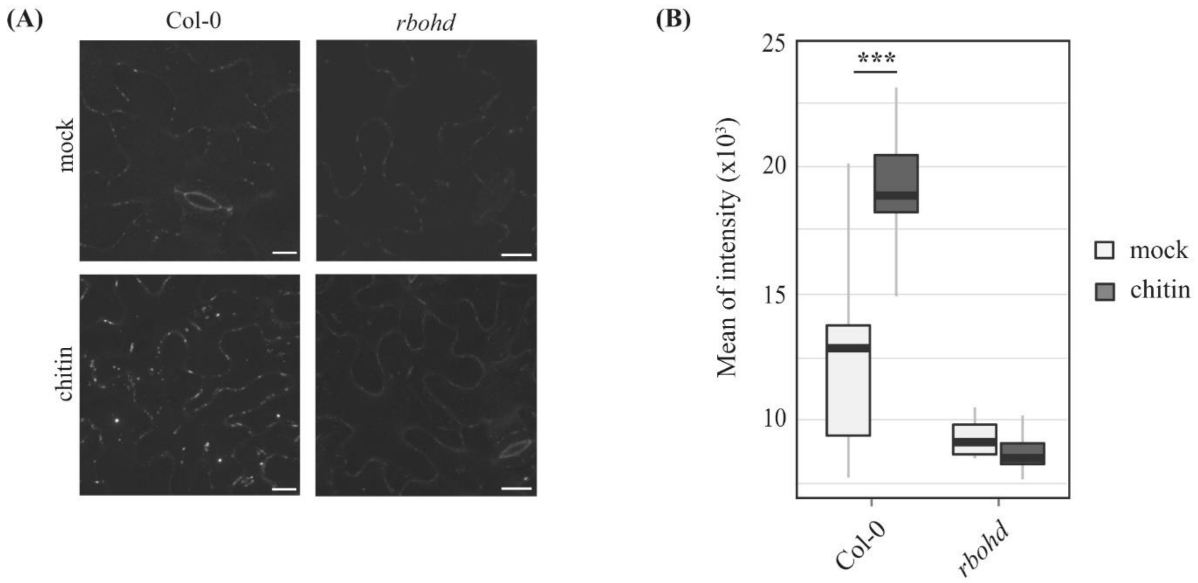

Figure S6. *rbohd* mutants do not deposit callose at plasmodesmata in response to chitin

(A) Confocal images of aniline blue stained plasmodesmal callose deposits in leaf tissue of 5- to 6-week-old leaves of Col-0 and *rbohd* mutant plants. Images were acquired 30 min post-infiltration with water or chitin. Scale bars are 15 µm. Images for Col-0 are as presented in Fig. 1B. (B) Quantification of plasmodesmata-associated fluorescence of aniline blue stained callose using automated image analysis. In contrast to Col-0, the *rbohd* mutant does not show increased aniline blue fluorescence at plasmodesmata in response to chitin. This correlates with the flux phenotype and identifies that chitin-triggered plasmodesmata closure is caused by callose deposition at plasmodesmata. The fluorescence intensity is summarised in box-plots in which the line within the box marks the median, the box signifies the upper and lower quartiles, the minimum and maximum within 1.5 × interquartile range. Data was analysed by pairwise Students' t-test comparing mock to chitin treated tissue within genotypes ($n \geq 31$, ***p-value < 0.001). Data for Col-0 is as presented in Fig. 1B.

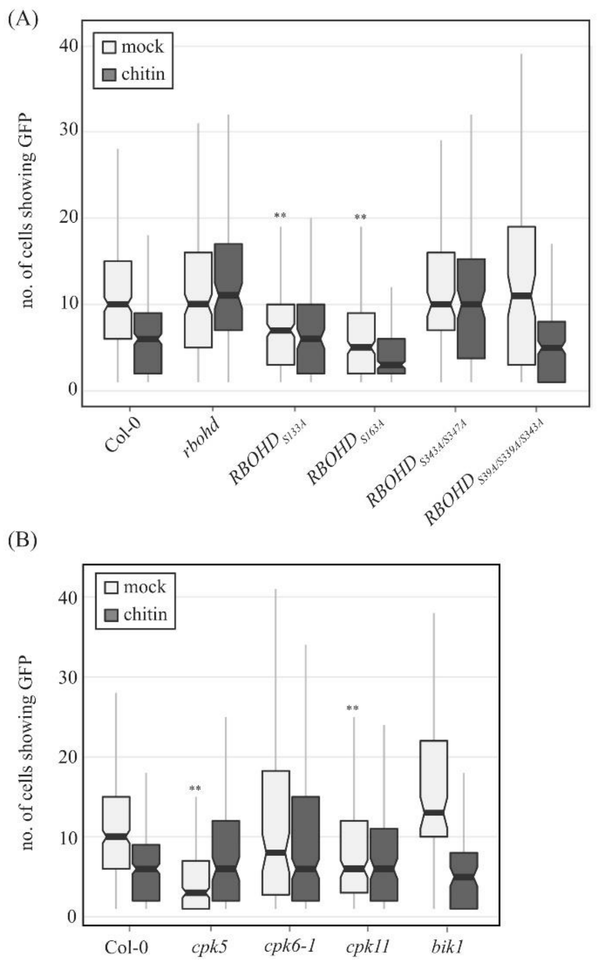

Figure S7. Raw, non-normalised data from bombardment experiments represented in Figure 5.

A. Analysis of non-normalised, microprojectile bombardment data presented in Figure 5A shows that *RBOHD*$_{S133A}$ and *RBOHD*$_{S163A}$ both exhibit reduced GFP flux from cell-to-cell in control conditions while other mutant variants of *RBOHD* show no different to wild type plants. B. Microprojectile bombardment data as described in Figure 5C. Analysis of non-normalised data shows that *cpk5* mutant has reduced GFP flux from cell-to-cell in control conditions and that chitin treatment increases GFP flux. *cpk5* and *cpk11* both show decreased GFP flux between cells in control conditions. The data is summarised in box-plots in which the line within the box marks the median, the box signifies the upper and lower quartiles, the whiskers represent the minimum and maximum within 1.5 × interquartile range. Notches represent approximate 95% confidence intervals. Number of bombardment sites (n) counted is indicated in Figure 1A. Asterisks indicate statistical significance compared to control conditions (***p*-value < 0.01).

Table S1. FRET efficiencies of LYK4-GFP in the presence and absence of the acceptor-RFP. The mean of FRET efficiency is expressed in percentage (%). PM, plasma membrane; PD, plasmodesmata; SE, standard error.

Localisation	Donor	Acceptor	Treatment	Additional Construct	FRET efficiency (%)	SE
PM	LYK4-GFP	_	_	_	_	_
PM	LYK4-GFP	LYK5-RFP	_	_	10.65	0.02
PM	LYK4-GFP	BRI1-RFP	_	_	3.76	0.43
PM	LYK4-GFP	_	chitin	_	2.05	0.46
PM	LYK4-GFP	LYK5-RFP	chitin	_	7.20	0.53
PM	LYK4-GFP	BRI1-RFP	chitin	_	2.74	0.56
PD	LYK4-GFP	_	_	Citrine-LYM2	_	_
PD	LYK4-GFP	LYK5-RFP	_	Citrine-LYM2	2.50	0.55
PM	LYK4-GFP	_	_	Citrine-LYM2	_	_
PM	LYK4-GFP	LYK5-RFP	_	Citrine-LYM2	4.69	0.55

Table S2. Primer sequences used in RT-PCR analysis of *LYSM-RK*s.

**Gene**	**Primer Sequences (5’-3’)**
ACTIN2	F: ATGGCTGAGGCTGATGATATTCAAC<br>R: CCAGGAATCGTTCACAGAAAATGTTTC
CERK1	F: ATGAAGCTAAAGATTTCTCTAATCGCTCCG<br>R: GTCAGTCTTATGTCCGGCCGGTAG
LYK2	F: ATGGCTGTTTCAGTTAGTAAGC<br>R: CCTTTGGTAAAGAAGAGTAGTATAATAG
LYK3	F: GCAAAGAGTGGTAGTCATGTGCC<br>R: GTGGTCTAGTCCAAGGAAGATAA
LYK4	F: CCACAATCGGTTTCTCCTCCTCCATTGTC<br>R: CTACGCAGAACTGGGAAGAATCGTCGTAC
LYK5	F: TTCTGGTCTCAACCACCGTAC<br>R: CATCCGTCTCTCAGGTTTCTG

Table S3: n values for experiments with unequal sample sizes. For bombardment experiments n is the number of bombardment sites counted; for callose staining, PD index, and anisotropy experiments n is the number of images analysed; for FLIM and FRAP experiments n is the number of single experiments; for ROS assays n is the number of seedlings (Fig 5B) or leaf discs (Figure S5).

**Bombardment**	**n**	**Figure**
Col-0 control	303	1A
Col-0 chitin	301	1A
lym2-1 control	101	1A
lym2-1 chitin	133	1A
lyk3 control	305	1A
lyk3 chitin	326	1A
lyk4 control	142	1A
lyk4 chitin	84	1A
lyk5 control	123	1A
lyk5 chitin	84	1A
rbohd control	212	5A
rbohd chitin	121	5A
bik1 control	93	5C
bik1 chitin	89	5C
S343A/S347A control	114	5A
S343A/S347A chitin	120	5A
S39A/S339A/S343A control	111	5A
S39A/S339A/S343A chitin	211	5A
S163A control	225	5A
S163A chitin	225	5A
S133A control	227	5A
S133A chitin	121	5A
cpk6-1 control	104	5C
cpk6-1 chitin	274	5C
cpk5 control	220	5C
cpk5 chitin	120	5C
cpk11-2 control	179	5C
cpk11-2 chitin	118	5C
Col-0 control	230	S5A
Col-0 H2O2	85	S5A
lym2-1 control	101	S5A
lym2-1 H2O2	91	S5A
**Callose Staining**		
Col-0 control	59	1C
Col-0 chitin	51	1C

lym2-1 control	40	1C
lym2-1 chitin	43	1C
lyk4 control	48	1C
lyk4 chitin	31	1C
lyk5 control	49	1C
lyk5 chitin	48	1C
cerk1-2 control	49	1C
cerk1-2 chitin	54	1C
rbohd control	37	S6B
rbohd chitin	45	S6B
**Cit-LYM2 PD Index**		
control	31	4B
chitin	31	4B
+ LYK4-RFP chitin	18	4B
+ LYK5-RFP chitin	18	4B
lym2/LYM2pro::Cit-LYM2 control	16	S4
lym2/LYM2pro::Cit-LYM2 chitin	16	S4
**Anisotropy**		
GFP	11	4C
Cit-LYM2 PM	21	4C
Cit-LYM2 PD	21	4C
**FRAP**		
Cit-LYM2 chitin PD	43	3D
Cit-LYM2 chitin PM	45	3D
Cit-LYM2 water PD	46	3D
Cit-LYM2 water PM	52	3D
LYK4-RFP chitin PM	52	3D
LYK4-RFP water PM	60	3D
LYK5-RFP chitin PM	50	3D
LYK5-RFP water PM	50	3D
**FLIM**		
LYK4-GFP	110	3B
LYK4-GFP+LYK5-RFP	71	3B
LYK4-GFP+ BRI1-RFP	39	3B
LYK4-GFP+ CHITIN	29	3B
LYK4-GFP+ LYK5-RFP+ CHITIN	40	3B
LYK4-GFP+ BRI1-RFP+ CHITIN	19	3B
LYK4-GFP + Cit-LYM2 PM	44	3C
LYK4-GFP + LYK5-RFP + Cit-LYM2 PM	38	3C
LYK4-GFP + Cit-LYM2 PD	27	3C
LYK4-GFP + LYK5-RFP + Cit-LYM2 PD	33	3C

**ROS assay**		
Col-0 water	23	5B
Col-0 chitin	24	5B
rbohd water	24	5B
rbohd chitin	21	5B
S133A water	21	5B
S133A chitin	21	5B
S163A water	20	5B
S163A chitin	19	5B
S343A/S347A water	20	5B
S343A/S347A chitin	19	5B
S39A/S339A/S343A water	23	5B
S39A/S339A/S343A chitin	24	5B
cpk6-1 water	21	5B
cpk6-1 chitin	20	5B
cpk11-2 water	24	5B
cpk11-2 chitin	23	5B
cerk1-2 water	24	5B
cerk1-2 chitin	23	5B
Agrobacterium only control	24	S5B
Agrobacterium only chitin	16	S5B
Cit-LYM2 control	33	S5B
Cit-LYM2 chitin	28	S5B
LYK4-RFP control	15	S5B
LYK4-RFP chitin	16	S5B
LYK5-RFP control	16	S5B
LYK5-RFP chitin	10	S5B

Supporting References

1. Faulkner C, et al. (2013) LYM2-dependent chitin perception limits molecular flux via plasmodesmata. *Proc Natl Acad Sci U S A* 110(22):9166–9170.
2. Bücherl CA, et al. (2017) Plant immune and growth receptors share common signalling components but localise to distinct plasma membrane nanodomains. *eLife* 6. doi:10.7554/eLife.25114.
3. Yoo SD, Cho YH, Sheen J (2007) Arabidopsis mesophyll protoplasts: a versatile cell system for transient gene expression analysis. *Nat Protoc* 2(7):1565–1572.
4. Xu B, et al. (2017) A calmodulin-like protein regulates plasmodesmal closure during bacterial immune responses. *New Phytol* 215(1):77–84.
5. Weidtkamp-Peters S, Stahl Y (2017) The Use of FRET/FLIM to Study Proteins Interacting with Plant Receptor Kinases. *Methods Mol Biol* 1621:163–175.
6. Spira F, et al. (2012) Patchwork organization of the yeast plasma membrane into numerous coexisting domains. *Nat Cell Biol* 14(6):640–648.
7. Martiniere A, et al. (2012) Cell wall constrains lateral diffusion of plant plasma-membrane proteins. *Proc Natl Acad Sci U S A* 109(31):12805–12810.
8. Faulkner C, Bayer EM (2017) Isolation of Plasmodesmata. *Methods Mol Biol* 1511:187–198.
9. Ruxton GD, Neuhäuser M (2013) Improving the reporting of P-values generated by randomization methods. *Methods Ecol Evol* 4(11):1033–1036.
10. Lenth R, Singmann H, Love J, Buerkner P, Herve M (2019) emmeans: Estimated Marginal Means, aka Least-Squares Means. R package version 1.3.3. Available at: https://cran.r-project.org/package=emmeans.
